# Supplementary material for: DOME Registry: implementing community-wide recommendations for reporting supervised machine learning in biology
Source: Gigascience. 2024 Dec 11;13:giae094. doi: 10.1093/gigascience/giae094 (PMC11633452; doi:10.1093/gigascience/giae094)
Supplement: giae094_Response_to_Reviewer_Comments_Original_Submission [file giae094_response_to_reviewer_comments_original_submission.pdf]

Author's Response To Reviewer Comments

Padova, 21 October 2024
Dear Editor,
We are submitting the revised version of our manuscript “DOME Registry: Implementing community-wide recommendations for reporting supervised machine learning in biology”, by Attafi, Clementel, et al.
Please find below our point-by-point response to the Reviewers’ comments in the following pages. We modified the manuscript in order to incorporate all the suggestions received. We are confident that they significantly improved the comprehension and reliability of our work.
We are very thankful to the reviewers for their effort in reading our manuscript and their constructive suggestions which have greatly improved the quality of our manuscript.
Yours faithfully,
Silvio C. E. Tosatto
Full Professor of Bioinformatics
Department of Biomedical Sciences
University of Padova

Response to Reviewer 1:

We would like to thank the reviewer for their positive comments and for appreciating our work.

QUERY:
1)It would be good to provide a mirroring or synchronization mechanism to allow users to establish mirror sites of DOME that can synchronize your site every week/month. This is because internet bandwidth is still an issue in some places of this world. Maybe, data packages that can be downloaded is an acceptable form for some
people. This is only a suggestion. If the authors feel this is too much work or not necessary, just ignore this.

RESPONSE:
On the About page, we provide an example of a DOME-registry mirror and a link to our GitHub repository with the steps for creating a local mirror. Please see URLs:
https://registry.dome-ml.org/about
https://github.com/BioComputingUP/dome-registry

QUERY:
2)Some instructions with screenshots on how to use the sites should be briefly included in the paper, if there is no length restriction.

RESPONSE:
Thanks for this suggestion. In the revised version of the manuscript, we have included a new figure (Figure 2) with screenshots of the browse and entry page, as well as a detailed explanation.
