# Supplementary material for: DOME Registry: implementing community-wide recommendations for reporting supervised machine learning in biology
Source: Gigascience. 2024 Dec 11;13:giae094. doi: 10.1093/gigascience/giae094 (PMC11633452; doi:10.1093/gigascience/giae094)
Supplement: giae094_Response_to_Reviewer_Comments_Revision_1 [file giae094_response_to_reviewer_comments_revision_1.pdf]

Author's Response To Reviewer Comments

We have included the additional sections as requested.
Please note that I was unable to find ROR entries for two funders -- see comment in manuscript Doc file.
